
JOURNAL INFORMATION
==============================
NLM Title Abbreviation: ScientificWorldJournal
Journal ID: ScientificWorldJournal
NLM Journal ID: 101131163
Journal ID: TSWJ

The Scientific World Journal

ISSN: 2356-6140
EISSN: 1537-744X
Publisher: Wiley

ARTICLE INFORMATION
==============================
PMCID: PMC6084176
PMID: 30147523
DOI: 10.1100/tsw.2001.174
Article ID: 398453
Article version: 1
Subjects: Short Report

Identification and Characterization of a Minimal Apoptotic Domain from p53

Ryan Kevin M. *
Vousden Karen H.
Regulation of Cell Growth Laboratory, NCI-FCRDC, P.O. Box B, Frederick, MD 21702, USA
*Kevin M. Ryan: ryank@mail.ncifcrf.gov
Publication date: 2001
Electronic publication date: 2001 Nov 15
Volume: 1
First page: 57
Last page: 57
Copyright: Copyright © 2001 Kevin M. Ryan and Karen H. Vousden.
Copyright year: 2001
License: This is an open access article distributed under the Creative Commons Attribution License, which permits unrestricted use, distribution, and reproduction in any medium, provided the original work is properly cited.
License URL: https://creativecommons.org/licenses/by/3.0/

==============================
Miami *Nature Biotechnology* Short Reports
*TheScientificWorld* (2001) 1(S3), 57SR
ISSN 1532-2246; DOI 10.1100/tsw.2001.174

### IDENTIFICATION AND CHARACTERIZATION OF A MINIMAL APOPTOTIC DOMAIN FROM P53

Kevin M. Ryan* and Karen H. Vousden

Regulation of Cell Growth Laboratory, NCI-FCRDC, P.O. Box B, Frederick, MD 21702
* ryank@mail.ncifcrf.gov

**INTRODUCTION.** The p53 tumor suppressor gene is the most frequently mutated gene in human cancer. Activation of p53 can cause either a transient growth arrest or apoptosis. The fact that in some cell types oncogenic transformation converts the p53 response from growth arrest to apoptosis provides a strong basis for exploiting p53 as a therapeutic target.

While transcriptional activation is considered the mechanism by which p53 mediates growth arrest, transcriptionally dependent and transcriptionally independent pathways have been implicated in p53's apoptotic response (1).

**METHODS.** To further investigate the transcriptionally independent apoptotic functions of p53, we decided to search for a minimal apoptotic domain within the protein. To obtain equal expression of different portions of the p53 protein, we fused all the mutants generated to GFP. These fusion proteins were then expressed in cells and assessed for there ability to induce gene expression by immunoblotting and reporter gene assays, and for their ability to induce programmed cell death by flow cytometry.

**RESULTS.** Similar to a previously described mouse mutant (2), we show here that a fragment containing the first 210 amino acids of human p53 (tr210) is similar to wild-type p53 in that it can induce considerable apoptosis when fused to GFP. However, in contrast to the wild-type GFP fusion protein, tr210-GFP was found to be completely incapable of transactivating the gene for the cyclin dependent kinase inhibitor, $p21^{waf-1/cip-1}$, and the pro-apoptotic target genes Bax, IGF-BP3 or PIG-3. This mutant is, however, similar to wild-type p53, in that it requires NF-κB to induce apoptosis and co-operates with TNFα in inducing programmed cell death. Most importantly, we found that the apoptotic activity of tr210 was not affected by the expression of a tumor-derived mutant of p53, which has been shown previously to repress the activity of wild-type p53 (3). Also, further refinement of this truncation revealed that only 37 amino acids from p53 were sufficient to induce as much apoptosis as tr210.

**DISCUSSION.** While further studies to investigate the potential differences in the apoptotic pathways engaged by these transcriptionally independent mutants and wild-type p53 should prove rewarding, it is the identification of such a small apoptotic domain from p53 which is undoubtedly the most exciting. This discovery opens up the possibility of mimicking the apoptotic activity of p53 with a drug that may then specifically target transformed cells. In this regard, it is also an important observation, that unlike wild-type p53, the activity of tr210 is not inhibited by expression of mutant p53, indicating that it has the advantage over therapeutic strategies involving full length p53, in that will probably kill tumor cells irrespective of endogenous p53 status.

**REFERENCES.**

1. Bates, S. and Vousden, K.H. (1999) Cell. Mol. Life Sci. 55, 28-37
2. Haupt, Y., Rowen, S., Shaulian, E., Vousden, K.H., and Oren, M. (1995) Genes Dev. 9, 2170-2183
3. Aurelio, O.N., Kong, X.-T., Gupta, S., and Stanbridge, E.J. (2000) Mol. Cell. Biol. 20, 770-778
